# B-lymphocyte stimulator/a proliferation-inducing ligand heterotrimers are elevated in the sera of patients with autoimmune disease and are neutralized by atacicept and B-cell maturation antigen-immunoglobulin

**DOI:** 10.1186/ar2959

**Published:** 2010-03-19

**Authors:** Stacey R Dillon, Brandon Harder, Kenneth B Lewis, Margaret D Moore, Hong Liu, Thomas R Bukowski, Nels B Hamacher, Megan M Lantry, Mark Maurer, Cecile M Krejsa, Jeff L Ellsworth, Susan Pederson, Keith B Elkon, Mark H Wener, Maria Dall'Era, Jane A Gross

**Affiliations:** 1 Preclinical Research and Development, ZymoGenetics, Inc., 1201 Eastlake Ave East, Seattle, WA 98102, USA; 2 Division of Rheumatology, School of Medicine, University of Washington, 1959 NE Pacific Street, Box 356428, Seattle, WA 98195-6428, USA; 3 Division of Rheumatology, Department of Medicine, University of California, San Francisco, 533 Parnassus Avenue, Box 0633, San Francisco, CA 94143-0633, USA

## Abstract

**Introduction:**

B-lymphocyte stimulator (BLyS) and a proliferation-inducing ligand (APRIL) are members of the tumor necrosis factor (TNF) family that regulate B-cell maturation, survival, and function. They are overexpressed in a variety of autoimmune diseases and reportedly exist *in vivo *not only as homotrimers, but also as BLyS/APRIL heterotrimers.

**Methods:**

A proprietary N-terminal trimerization domain was used to produce recombinant BLyS/APRIL heterotrimers. Heterotrimer biologic activity was compared with that of BLyS and APRIL in a 4-hour signaling assay by using transmembrane activator and CAML interactor (TACI)-transfected Jurkat cells and in a 4-day primary human B-cell proliferation assay. A bead-based immunoassay was developed to quantify native heterotrimers in human sera from healthy donors (n = 89) and patients with systemic lupus erythematosus (SLE; n = 89) or rheumatoid arthritis (RA; n = 30). Heterotrimer levels were compared with BLyS and APRIL homotrimer levels in a subset of these samples.

**Results:**

The recombinant heterotrimers consisted mostly of one BLyS and two APRIL molecules. Heterotrimer signaling did not show any significant difference compared with APRIL in the TACI-Jurkat assay. Heterotrimers were less-potent inducers of B-cell proliferation than were homotrimeric BLyS or APRIL (EC_50_, nMol/L: BLyS, 0.02; APRIL, 0.17; heterotrimers, 4.06). The soluble receptor fusion proteins atacicept and B-cell maturation antigen (BCMA)-immunoglobulin (Ig) neutralized the activity of BLyS, APRIL, and heterotrimers in both cellular assays, whereas B-cell activating factor belonging to the TNF family receptor (BAFF-R)-Ig neutralized only the activity of BLyS. In human sera, significantly more patients with SLE had detectable BLyS (67% versus 18%; *P *< 0.0001), APRIL (38% versus 3%; *P *< 0.0002), and heterotrimer (27% versus 8%; *P *= 0.0013) levels compared with healthy donors. Significantly more patients with RA had detectable APRIL, but not BLyS or heterotrimer, levels compared with healthy donors (83% versus 3%; *P *< 0.0001). Heterotrimer levels weakly correlated with BLyS, but not APRIL, levels.

**Conclusions:**

Recombinant BLyS/APRIL heterotrimers have biologic activity and are inhibited by atacicept and BCMA-Ig, but not by BAFF-R-Ig. A novel immunoassay demonstrated that native BLyS/APRIL heterotrimers, as well as BLyS and APRIL homotrimers, are elevated in patients with autoimmune diseases.

## Introduction

B-lymphocyte stimulator (BLyS)-also called B cell-activating factor belonging to the tumor necrosis factor family (BAFF)-and a proliferation-inducing ligand (APRIL) are members of the tumor necrosis factor (TNF) family and are important regulators of B-cell maturation, survival, and function [1,2]. The TNF ligands generally form trimeric structures composed of three monomers [3]. Heterotrimers of BLyS and APRIL have also been shown to exist *in vivo*, and a higher-order oligomer of BLyS homotrimers has been reported [4-6].

BLyS homotrimers bind to the B cell-expressed receptors transmembrane activator and CAML interactor (TACI), B cell-maturation antigen (BCMA), and BAFF receptor (BAFF-R), whereas APRIL homotrimers bind to TACI, BCMA, and proteoglycans [7,8]. The binding of BLyS and APRIL to these receptors activates specific TNF receptor-associated factors (TRAFs), which regulate signal transduction in B cells. The interaction with TRAFs induces the nuclear factor (NF)-κB signaling pathway, which plays a pivotal role in regulating diverse aspects of immune function, including mediating inflammatory responses and facilitating adaptive immunity [9-11]. The binding of BLyS and APRIL to TACI, BCMA, and BAFF-R receptors also triggers the upregulation or downregulation of members of the Bcl-2 family of proteins, which are involved in cell death, proliferation, survival, and cell-cell interactions [12]. It has been proposed that signaling through TACI in mature B cells or plasmablasts requires higher-order BLyS oligomers or the cross-linking of APRIL through its binding to proteoglycans, whereas BAFF-R and TACI on primary B cells can bind and respond to all forms of BLyS [4,8,13].

BLyS and APRIL are overexpressed in the sera of patients with a wide variety of autoimmune disorders, including systemic lupus erythematosus (SLE) [14,15]. In patients with rheumatoid arthritis (RA), BLyS and APRIL are overexpressed in the synovial fluid as well as in the sera [6,16]. Preliminary data suggest that BLyS/APRIL heterotrimers also are elevated in patients with various autoimmune conditions [6]. In light of their roles in B-cell function and these clinical data, BLyS and APRIL are targets for novel treatments for autoimmune diseases.

Atacicept is a fully human recombinant fusion protein comprising the extracellular portion of the TACI receptor linked to an Fc domain of immunoglobulin (Ig)G. Atacicept modulates B cells by neutralizing BLyS and APRIL activity and is in clinical development for the treatment of SLE and RA [17,18]. As BLyS/APRIL heterotrimers may also be elevated in patients with autoimmune diseases, it is important to determine whether these heterotrimers play a particular biologic role, and if therapies targeting BLyS and APRIL will also neutralize BLyS/APRIL heterotrimers. This study investigated the *in vitro *activity of recombinant heterotrimers in cell-signaling and proliferation assays, and the ability of the soluble B cell-expressed receptors atacicept, BCMA-Ig, and BAFF-R-Ig to neutralize heterotrimer activity. A bead-based immunoassay was developed for BLyS/APRIL heterotrimers, and endogenous levels of heterotrimers in the sera of healthy donors and patients with SLE or RA were measured and compared with BLyS and APRIL homotrimer levels in a subset of the same samples.

## Materials and methods

### Production of recombinant BLyS and APRIL homotrimers and BLyS/APRIL heterotrimers

BLyS and APRIL homotrimers were generated as previously described [19,20]. Recombinant BLyS/APRIL heterotrimers were produced by using a proprietary *N*-terminal trimerization domain [21]. The Flag-zippered (zz) 12.6 form of APRIL [21] and the Hisx6-zz12.6 form of BLyS were made by overlap polymerase chain reaction (PCR) of human APRIL [amino acids 110-250] template and human BLyS [amino acids 141-285] template, respectively. The assembled cDNA was inserted by homologous recombination into the vectors pZMP21 [22] and pZMP41z, respectively, downstream of the optimized tissue plasminogen activator (otPA) leader sequence. These vectors were transfected into protein-free media-adapted Chinese hamster ovary (CHO) DXB11 cell suspension by electroporation, and the cells were selected for growth in methotrexate- and copper-chelated antibiotic (Zeocin)-containing medium. Methotrexate- and Zeocin-resistant cells were stained with fluorescein isothiocyanate-anti-CD8 and phycoerythrin-anti-CD4, and CD8^+^/CD4^+ ^cells selected by fluorescence-activated cell sorting. Batches of the BLyS/APRIL heterotrimer were produced in a 10-L WAVE bioreactor. Both BLyS and APRIL were quantified by Western blot with anti-His and anti-Flag antibodies, respectively, as described later. The expression levels of BLyS NH6 zz12.6 and APRIL NF zz12.6 were approximately 10 mg/L and 33 mg/L, respectively.

### Purification of recombinant BLyS/APRIL heterotrimers

Heterotrimers were purified from CHO-conditioned media after buffer exchange to phosphate-buffered saline (PBS) (pH 7.4) by ultrafiltration/diafiltration and loading onto an immobilized metal affinity chromatography NI-NTA His Bind Superflow Column (Novagen, Gibbstown, NJ, USA). A heparin affinity column (HAC), Heparin AF-650M (Tosoh Bioscience, Montgomeryville, PA, USA), was used to resolve the heterotrimer from Flag-zz12.6 APRIL via NaCl gradient elution. The HAC eluate was concentrated to < 8 ml and injected over a size-exclusion chromatography (SEC) Superdex 200 Prep Grade Column (GE Healthcare, Piscataway, NJ, USA).

### Generation of non-tagged recombinant BLyS/APRIL heterotrimers

Non-tagged heterotrimers were produced by using a limited proteolysis strategy with trypsin to cleave the Flag and His tags from the zz12.6 heterotrimers. A HAC (as earlier), was used to resolve non-tagged heterotrimer from undigested products by NaCl gradient elution. The HAC eluate was concentrated to < 3 ml and injected over an SEC column (as earlier). The SEC eluate was incubated and rocked slowly overnight with 1 ml of anti-Flag agarose resin (Sigma, St Louis, MO, USA). The resin was separated from solution via 0.22-μm filtration. N-terminal sequence analysis, SEC with multiangle light scattering (SEC-MALS), and Western blot analyses were consistent with both tags having been removed.

### Protein detection

APRIL or BLyS protein samples were visualized by using nonreducing sodium dodecylsulfate polyacrylamide gel electrophoresis (SDS-PAGE). Analysis with Western blotting was performed by using standard methods. The anti-APRIL blot comprised anti-Flag horseradish peroxidase (HRP) for the Flag-zz12.6 form, and anti-APRIL polyclonal antibody (pAb) followed by donkey anti-rabbit IgG-HRP for the trypsinized form of APRIL. The anti-BLyS blot comprised anti-6x His HRP for the Hisx6-zz12.6 form of BLyS, and anti-BLyS pAb followed by donkey anti-rabbit IgG-HRP for the trypsinized form of BLyS. The molecular mass of the heterotrimers was confirmed by SEC-MALS mass distribution LS/UV/RI 3-detector analysis.

### Binding kinetics and affinity studies

The binding affinities and kinetics of BLyS (average values were determined from three distinct lots of protein), APRIL (six lots), and heterotrimers (one lot) for the receptor-Fc fusion proteins (atacicept, BCMA-Ig, and BAFF-R-Ig) were assessed with Biacore surface plasmon resonance studies by using a Biacore 3000 analyzer (GE Healthcare) equipped with Biacore Control, Evaluation, and Simulation software version 3.2.

Atacicept, BCMA-Ig, and BAFF-R-Ig were covalently immobilized onto a Biacore CM4 sensor chip. Binding affinity studies were performed at 25°C with a 50 μl/min flow rate for varying ligand concentrations. Serial 1:2 dilutions of each ligand from ~0.05 to 20 nMol/L were made in analysis buffer (20 mmol/L sodium phosphate, 150 mmol/L NaCl, 0.05% polysorbate 20, pH 7.5). Dissociation constant (*K*_D_) values were determined from the kinetic rate constants (*k*_*a *_and *k*_*d*_). The binding curves were processed by double referencing and were globally fitted to a 1:1 binding model. The stoichiometry of binding was not determined.

### Biologic activity assays

A 4-hour signaling assay was performed by using TACI-transfected Jurkat cells carrying an NF-κB/luciferase reporter gene (KZ142). TACI/KZ142-Jurkat cells (1 × 10^5 ^cells/well) were incubated at 37°C for 4-6 hours with recombinant BLyS, APRIL, or heterotrimer in a total well volume of 100 μl in complete RPMI-1640 media without phenol red, and in the absence or presence of atacicept, BCMA-Ig, or BAFF-R-Ig in concentrations ranging from 0.001 to 100 nMol/L. After incubation, 100 μl/well of Steady-Glo reagent was added, and the plates were foil covered and agitated at room temperature for 10 minutes. The assay plate was then analyzed in a luminometer to measure luciferase activity.

For the human B cell-proliferation assay, B cells from two healthy donors were isolated from peripheral blood mononuclear cells by negative selection with the human B Cell Isolation Kit II from Miltenyi Biotec (Auburn, CA, USA), according to the manufacturer's instructions. Flow cytometry confirmed that they were > 97% pure (CD19^+^). The purified B cells (5 × 10^4 ^cells/well) were plated in a 96-well flat-bottomed plate pre-coated with 5 μg/ml anti-IgM monoclonal antibody (mAb) (Southern Biotech, Birmingham, AL, USA) in media containing 10 ng/ml recombinant human interleukin-4 (R&D Systems, Minneapolis, MN, USA) and BLyS, APRIL, or heterotrimer at concentrations ranging from 0.001 to 100 nMol/L. The plates were then incubated for 4 days at 37°C, and proliferation was determined by^3^H-thymidine incorporation assay. To determine the relative neutralization with the soluble receptors, atacicept, BCMA-Ig, or BAFF-R-Ig, the assay was set up as previously described by using 50% effective concentrations (EC_50_) of BLyS, APRIL, and heterotrimers (1, 3, and 10 nMol/L, respectively). Starting with a 300-fold molar excess, 1:4 serial dilutions of atacicept, BCMA-Ig, or BAFF-R-Ig were then added to the wells, and the assay run as described earlier. Fifty percent inhibition concentration (IC_50_) values were determined for the inhibition of ligand activity by each soluble receptor by using purified B cells from a third donor.

Please see Supplemental Methods in Additional file 1 for more-detailed descriptions of the generation, purification, and characterization of the recombinant heterotrimers, and of the binding affinity and biologic activity assays.

### Generation of anti-APRIL and anti-BLyS monoclonal antibodies

Anti-human APRIL mAbs were generated from BALB/c mice immunized with 50 μg of recombinant Flag-zz12.6 APRIL in combination with Ribi-CWS adjuvant (Corixa-Sigma, St Louis, MO, USA) every 2 weeks over an 8-week period. Serum titers were determined for the presence of anti-APRIL antibodies. The mice with the most significant anti-APRIL serum titers were immunized a final time. Four days later, the spleen and lymph nodes of the mice were harvested and fused to mouse myeloma P3-X63-Ag8.653 cells (American Type Culture Collection) at a 1:1 lymphocyte/myeloma ratio with polyethylene glycol 1500 by using standard methods. Wells of the fusion plates were fed 3 times with a 70% replacement of media, and wells were assayed 10 and 12 days after the plating of the fusion for anti-APRIL antibodies. Anti-human BLyS mAbs were prepared as described earlier, except that 20 μg of baculovirus-produced His-zz12.6 BLyS was used for the initial immunizations followed by 10-μg maintenance boosts. Antibody purifications were performed from hybridoma supernatant by Protein G (GE Healthcare) affinity chromatography followed by pH elution.

### Heterotrimer immunoassay

After pre-wetting and blocking a Luminex plate with assay buffer (PBS with 0.05% Tween 20, 1% bovine serum albumin), 5 × 10^3 ^anti-APRIL capture mAb-coated beads/well were added to the plate in 25 μl/well assay buffer. To this, 25 μl of standard plus 25 μl of normal human serum (pre-screened for low heterotrimer levels) or 25 μl of sample serum plus 25 μl of assay buffer was added. The plate was incubated on a shaker for 1 hour at room temperature, and then washed twice with 100 μl of assay buffer. Biotinylated anti-BLyS antibody (25 μl/well) was added, and the plate was incubated again as described earlier. Streptavidin-phycoerythrin (25 μl) was then added at 1:200 in assay buffer and incubated on a shaker for 30 minutes at room temperature. The plate was washed twice with 100 μl of assay buffer. Wells were then resuspended in 100 μl of assay buffer and analyzed by using a Luminex 100 machine (Luminex Corporation, Austin, TX, USA). The total assay time was 2 hours. The assay had a broad range (~100 pg/ml to 25 ng/ml) with an EC_50 _of ~6 ng/ml.

Luminex-based assays may vary in sensitivity due to differences in the capture-bead lots used. In this study, the heterotrimer assay data were generated by using two lots of beads with slightly different limits of quantitation (LOQs), 0.100 ng/ml versus 0.313 ng/ml. To make use of the combined dataset, the most conservative LOQ (0.313 ng/ml) was applied to all heterotrimer data, although some sample cohorts had reportable values below this concentration (see Supplemental Tables 1 and 2 in Additional file 2).

### Serum heterotrimer and BLyS and APRIL homotrimer levels in patients with autoimmune diseases

Serum samples from 30 patients with SLE and 30 patients with RA were obtained from the University of Washington (Seattle, WA, USA) serum repository. Dr. Dall'Era and Dr. Wofsy from the University of California, San Francisco (UCSF) (San Francisco, CA, USA) provided 59 serum samples from 47 patients with SLE and from nine healthy donors. Twenty of the UCSF serum samples from patients with SLE were collected from the same eight patients over time (two to four draws each, drawn a minimum of 1 month apart). All patients fulfilled the revised American College of Rheumatology classification criteria for SLE and RA. Patients were enrolled after obtaining their written informed consent by using a protocol approved by the Human Subjects Committee of the University of Washington and the Committee on Human Research at UCSF, respectively. Serum samples from an additional 80 healthy donors were collected at ZymoGenetics, Inc. (Seattle, WA, USA).

Serum samples from healthy donors (n = 89) and patients with SLE (n = 89) or RA (n = 30) were collected in ethylenediaminetetraacetic acid (EDTA) tubes and frozen at -80°C. Samples were thawed and assessed for native heterotrimer levels by using the heterotrimer immunoassay described earlier. BLyS and APRIL levels were measured in a subset of the serum samples from healthy donors (n = 40) and patients with SLE (n = 30).

BLyS levels were measured with enzyme-linked immunosorbent assay (ELISA), as described previously [18]. All samples were measured in triplicate. As assay performance criteria, a precision of 20% for the coefficient of variation in the patient samples was accepted. The LOQ was 0.78 ng/ml of BLyS in the serum. APRIL levels were determined by using an ELISA developed and validated at ZymoGenetics, Inc. (Seattle, WA, USA) [20]. The LOQ was 2 ng/ml of APRIL in the serum. Because of insufficient volume, one of 30 available SLE serum samples and one of 40 healthy donor samples could not be included for assessment in the APRIL ELISA.

### Statistical methods

All statistical analyses were performed by using Prism 4 for Windows (GraphPad Software, Inc., La Jolla, CA, USA). The proportions of serum samples with BLyS or APRIL homotrimer or BLyS/APRIL heterotrimer levels above the assay LOQ in patients with SLE or RA were compared with those for the healthy donor cohort by using Fisher's exact test. A value of *P *< 0.05 was considered to be statistically significant. To reduce the impact of data skewing, correlation analysis for BLyS, APRIL, and heterotrimers levels was assessed by using Spearman's rank correlation coefficient. For the correlation analysis and for data plotting, samples with serum ligand levels below the assay LOQ were assigned values equal to half of the LOQ for each assay (0.39, 1.0, and 0.156 ng/ml for BLyS, APRIL, and heterotrimer, respectively). Bivariate correlation plots were generated by using JMP software (SAS Institute, Inc.).

## Results

### Characterization of heterotrimers

**Figure 1:**
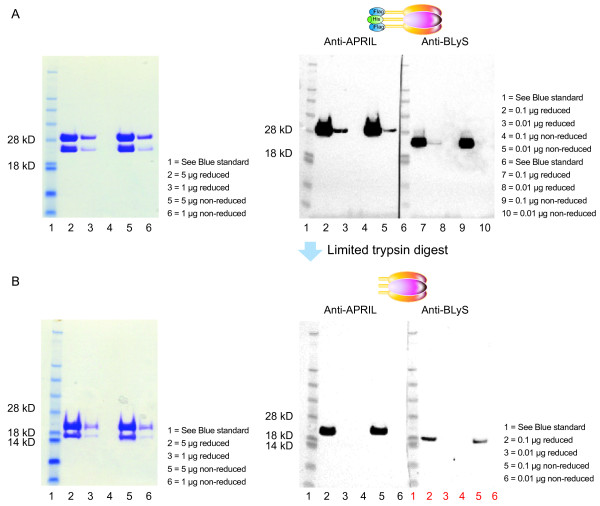
**Sodium dodecylsulfate polyacrylamide gel electrophoresis and Western blot analysis of (a) BLyS/APRIL heterotrimer and (b) trypsinized (nonzippered) heterotrimer**. APRIL, a proliferation-inducing ligand; BLyS, B-lymphocyte stimulator.

**Figure 2:**
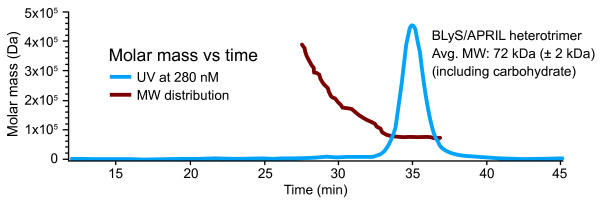
**Size-exclusion chromatography with multiangle light-scattering mass distribution of purified HT**. Red line, molecular weight species by static light scattering; blue line, BLyS/APRIL HT; APRIL, a proliferation-inducing ligand; BLyS, B-lymphocyte stimulator; HT, heterotrimer; MW, molecular weight.

The recombinant BLyS/APRIL heterotrimers were characterized by SDS-PAGE and Western-blot analyses (Figure 1). The molecular mass and purity of the heterotrimers were confirmed by SEC-MALS analysis (Figure 2). Because of unequal BLyS and APRIL expression plasmid efficiencies (see Materials and methods), the recombinant heterotrimers had a predominant stoichiometry of 2 APRIL to 1 BLyS (A_2_B), and thus consisted of only a small fraction of 1 APRIL to 2 BLyS (AB_2_) heterotrimers. The ratio of ~66.3% APRIL and 33.7% BLyS was identified by Western blot, and confirmed by SEC-MALS analysis.

The binding affinities of BLyS, APRIL, and heterotrimers for the soluble receptor fusion proteins atacicept, BCMA-Ig, and BAFF-R-Ig, were determined from one or two surface plasmon resonance experiments for each ligand. BLyS (n = 2) was the only ligand that exhibited binding to BAFF-R-Ig (K_D _= 0.02-0.07 nMol/L). BLyS (n = 2), APRIL (n = 2), and heterotrimers (n = 1) bound to atacicept (*K*_D _= 0.02 nMol/L [BLyS], 0.1-0.2 nMol/L [APRIL], and 0.4 nMol/L [heterotrimers]) and BCMA-Ig (*K*_D _= 0.3 nMol/L [BLyS], 0.0001-0.0003 nMol/L [APRIL], and 0.01 nMol/L [heterotrimers]).

### Biologic activity and neutralization of heterotrimers

**Figure 3:**
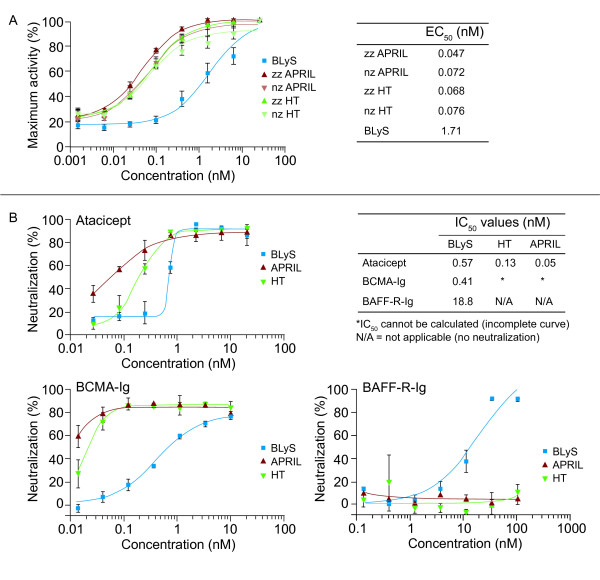
**(a) Biologic activity and (b) neutralization of BLyS/APRIL HTs compared with those of BLyS and APRIL in TACI-Jurkat proliferation assays**. APRIL, a proliferation-inducing ligand; BAFF-R, BAFF receptor; BCMA, B cell-maturation antigen; BLyS, B-lymphocyte stimulator; EC_50_, 50% effective concentration; HT, heterotrimer; IC_50_, 50% inhibition concentration; Ig, immunoglobulin; nz, trypsinized trimer without the "zipper" domain; TACI, transmembrane activator and CAML interactor; zz, trimer containing the "zipper" trimerization domain.

Heterotrimer signaling was similar to that of APRIL in the *in vitro *TACI-Jurkat assay (Figure 3a). Trypsinized versions of the heterotrimers and APRIL that lacked the proprietary "zipper" trimerization domain needed for their efficient expression were equally as active as the zz versions. This suggested that the zipper domain did not alter the biologic activity of these ligands in this assay. Atacicept and BCMA-Ig neutralized the activity of BLyS, APRIL, and heterotrimers in the TACI-Jurkat assay (Figure 3b). As expected, BAFF-R-Ig only neutralized the activity of BLyS.

**Figure 4:**
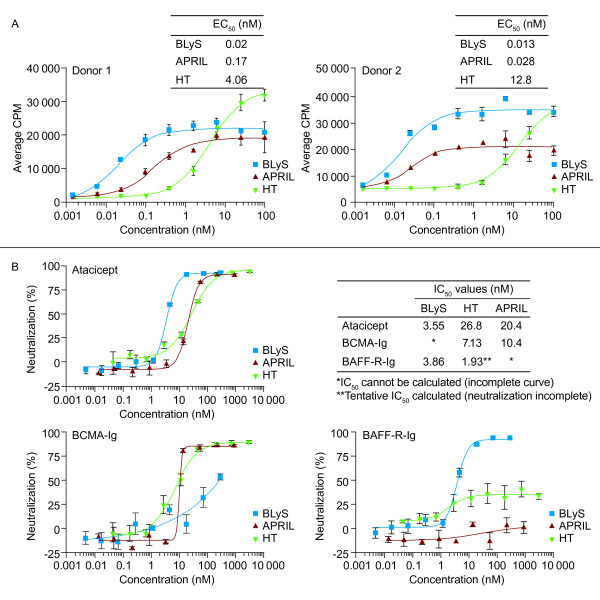
**(a) Biologic activity and (b) neutralization of BLyS/APRIL HTs compared with those of BLyS and APRIL in B cell-proliferation assays**. BLyS, APRIL, and heterotrimers were used at concentrations of 1 nMol/L, 3 nMol/L, and 10 nMol/L, respectively. APRIL, a proliferation-inducing ligand; BAFF-R, BAFF receptor; BCMA, B cell-maturation antigen; BLyS, B-lymphocyte stimulator; CPM, counts per minute; EC_50_, 50% effective concentration; HT, heterotrimer; IC_50_, 50% inhibition concentration; Ig, immunoglobulin; TACI, transmembrane activator and CAML interactor.

The heterotrimers were less-potent inducers of B-cell proliferation than were BLyS or APRIL, as evidenced by the higher EC_50 _values for heterotrimers than those of BLyS or APRIL in the primary human B cell-proliferation assay (Figure 4a). In the neutralization assay, BAFF-R-Ig inhibited BLyS but exhibited little to no inhibition of heterotrimer or APRIL activity on human B cells (Figure 4b).

### Heterotrimer immunoassay

**Figure 5:**
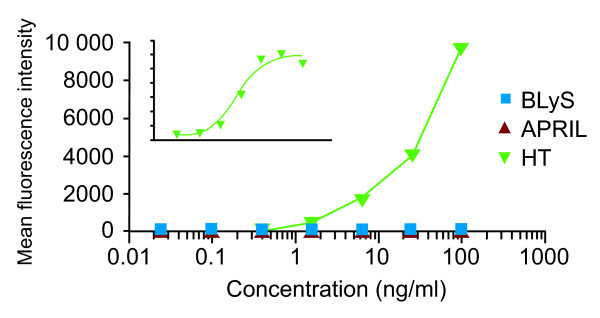
**Detection of BLyS/APRIL HTs by using a bead-based HT immunoassay**. The assay has a broad detection range (~25 ng/ml to ~100 pg/ml; see standard curve, inset), with an LOQ of ~100-313 pg/ml in the presence of serum (EC_50_~6 ng/ml). The average serum sample size required is 25 μl, and the total assay time is 2 hours. The assay does not detect BLyS or APRIL homotrimers. APRIL, a proliferation-inducing ligand; BLyS, B-lymphocyte stimulator; EC_50_, 50% effective concentration; HT, heterotrimer; LOQ, limit of quantitation.

To address whether endogenous heterotrimers are present in the sera of patients with autoimmune diseases, the recombinant heterotrimers were used as a standard to develop a bead-based immunoassay by using anti-APRIL capture mAb and fluorescence-labeled anti-BLyS detection mAb to quantify native heterotrimers in human sera. The bead-based assay used a recombinant protein heterotrimer that was heavily skewed toward A_2_B BLyS/APRIL trimers as a reference standard, but nevertheless, was also able to detect AB_2 _trimers. During the assay, beads conjugated with an anti-APRIL mAb were incubated with the test sample and washed, and then bead-bound heterotrimers were detected with a biotinylated anti-BLyS detection mAb. This format allowed the detection of both A_2_B and AB_2 _native heterotrimers. We confirmed that the new assay detected BLyS/APRIL heterotrimers but did not detect BLyS or APRIL homotrimers (Figure 5).

### Heterotrimer, BLyS, and APRIL levels in patients with autoimmune diseases

**Figure 6:**
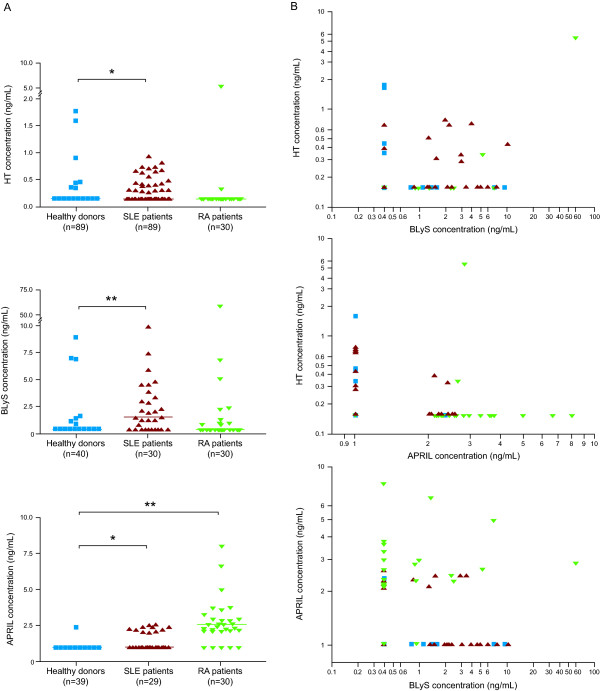
**Serum levels of BLyS, APRIL, and HTs in healthy donors and patients with autoimmune diseases**. **(a) **Serum concentrations of HTs (upper panel), BLyS (middle panel), and APRIL (lower panel) for each patient. Horizontal bars depict median values for each serum donor group: healthy donors (blue squares), patients with SLE (red triangles), and patients with RA (green inverse triangles). *P *values were determined by using Fisher's exact test. *P < 0.05, **P < 0.0001. **(b) **Bivariate plots showing serum levels of BLyS, APRIL, and HTs in healthy donors (blue squares), patients with SLE (red triangles), and patients with RA (green inverse triangles). For data plotting, samples with serum ligand levels below the LOQ were assigned values equal to half the LOQ for each assay (0.156, 0.39, and 1.0 ng/ml for HT, BLyS, and APRIL, respectively). APRIL, a proliferation-inducing ligand; BLyS, B-lymphocyte stimulator; HT, heterotrimer; LOQ, limit of quantitation; RA, rheumatoid arthritis; SLE, systemic lupus erythematosus.

The heterotrimer immunoassay and previously reported ELISAs for BLyS and APRIL were used to measure native heterotrimer, BLyS, and APRIL levels in serum samples from healthy donors and patients with SLE or RA (Figure 6a and Table 1). Significantly more patients with SLE had detectable heterotrimers in sera (27%, *P *= 0.0013) compared with healthy donors (8%), whereas detectable heterotrimer levels were found in only 7% (*P *= nonsignificant) of the samples from patients with RA (Table 1).

**Table 1:** Serum heterotrimer, BLyS, and APRIL levels in healthy donors, and patients with SLE or RA

	Heterotrimer		BLyS		APRIL	
			
Patients	Number of samples with > LOQ *n/N *(%)	Mean ± SD^a^ (ng/ml)	Number of samples with > LOQ *n/N *(%)	Mean ± SD^a^ (ng/ml)	Number of samples with > LOQ *n/N *(%)	Mean ± SD^a^ (ng/ml)
Healthy donors	7/89 (7.8)	0.83 ± 0.61	7/40 (17.5)	3.91 ± 3.47	1/39 (2.6)^b^	2.35 ± 0
SLE	24/89 (26.9)	0.52 ± 0.19	20/30 (66.7)	3.28 ± 2.35	11/29 (37.9)^b^	2.31 ± 0.18
RA	2/30 (6.7)	2.85 ± 3.55	10/30 (33.3)	8.07 ± 18.0	25/30 (83.3)	3.15 ± 1.44

^a^Mean concentration for serum samples with detectable ligand levels.

^b^In the subset of serum samples available, one sample from a healthy donor and one from a patient with SLE were not assayed because of insufficient volume.

LOQ, limit of quantitation; RA, rheumatoid arthritis; SD, standard deviation; SLE, systemic lupus erythematosus.

Analyses of BLyS and APRIL homotrimer levels were performed in a subset of the samples from healthy donors and patients with SLE, and for all of the samples from patients with RA (Figure 6a and Table 1). In sera from patients with SLE, significantly more samples had detectable BLyS (67%; *P *< 0.0001) and APRIL (38%; *P *< 0.0002) levels compared with the healthy donor cohort (18% (BLyS) and 3% (APRIL)) (Figure 6a and Table 1). Strikingly, although APRIL was detectable in most samples for patients with RA (83%; *P *< 0.0001) compared with healthy donors (3%), no statistically significant difference in detectable levels of BLyS was found in patients with RA (33%) compared with the healthy donor samples (18%) (Table 1). Within the samples containing detectable levels of the three ligands, no obvious differences were seen between the mean values of BLyS, APRIL, or heterotrimers levels in healthy donors compared with the mean values of each ligand in patients with SLE or RA, with the possible exception of elevated APRIL levels in patients with RA (Table 1). The mean detectable heterotrimers levels were generally similar to or lower than the BLyS and APRIL levels in the same patient cohorts (Table 1). Notably, in one sample from a patient with RA in which all three ligands were detectable, heterotrimer levels (5.37 ng/ml) exceeded those of APRIL (2.87 ng/ml), but not of BLyS (58.9 ng/ml).

A weak correlation was found between BLyS and heterotrimer levels (Spearman *r *= 0.2328, *P *< 0.02) in samples for which data for both ligands were available (Figure 6b, upper panel). No correlations were present between APRIL and heterotrimer levels or APRIL and BLyS levels (Figure 6b, middle and lower panels, respectively). Interestingly, in the 98 samples for which data on all three ligands were available, 43 had detectable levels of only one ligand: 22 with APRIL, 16 with BLyS, and five with heterotrimers. Two samples from patients with RA and one sample from a patient with SLE had detectable levels of all three ligands, whereas samples from six patients with SLE had detectable levels of both BLyS and heterotrimers. In contrast, levels were below the LOQ for all three ligands in the majority of samples from healthy donors for which data from all three assays were available (27 of 36, 75%) (Figure 6 and data not shown).

### Correlation between heterotrimer levels and disease activity markers for patients with SLE

A subset of the samples from patients with SLE used in the heterotrimer assay had previously been assessed for various markers of disease activity, including patient SLE Disease Activity Index (SLEDAI) scores at the time of serum draw, erythrocyte sedimentation rate (ESR), concentrations of anti-double stranded DNA (dsDNA) antibodies, and levels of the complement components C3 and C4. Although this information was available for a relatively small group of these samples (n = 36), the data were analyzed to seek any potential correlations between serum heterotrimer levels and other markers of SLE disease activity. The 36 samples from patients with SLE were separated into three groups determined by the heterotrimer levels found in each sample: undetectable, low, and high heterotrimer levels. The median heterotrimer concentration in the 27 positive samples was 0.227 ng/ml; all samples with heterotrimers levels above this value were assigned to the "high" heterotrimer group. Typically, as the SLE disease state worsens, patients' serum C3 and C4 levels decrease, whereas SLEDAI scores, anti-dsDNA antibody levels, and ESR all increase. We observed a trend suggesting that heterotrimer levels increase along with SLEDAI, anti-dsDNA antibodies, and possibly ESR (Supplemental Tables 1 and 2 in Additional file 2). C3 and C4 levels were also lower in samples with detectable heterotrimer levels, compared with those samples with undetectable heterotrimer levels.

## Discussion

In this study, recombinant BLyS/APRIL heterotrimers were produced by using a novel trimerization domain, purified, and extensively characterized. Their biologic activity was evaluated *in vitro*, and the effect of atacicept and other related soluble receptors on their activity was determined. A novel immunoassay was developed, and the occurrence of endogenous heterotrimers in healthy donors and patients with SLE and RA was demonstrated. Heterotrimer levels were compared in a subset of the patient samples with levels of homotrimeric BLyS and APRIL.

Several trimeric forms have been proposed for BLyS and APRIL. In principle, BLyS/APRIL heterotrimers could be any mix of stoichiometries (A_2_B or AB_2_). The heterotrimers produced for this study were predominantly A_2_B, and their biologic activities were more similar to APRIL than to BLyS. The A_2_B stoichiometry was obtained because of the characteristics of the two vectors that were used to express BLyS and APRIL in the production cell lines. *In vivo*, BLyS/APRIL heterotrimers are likely to be formed stochastically in cells in which both BLyS and APRIL are generated, and the relative production of these proteins may influence the proportion of A_2_B and AB_2 _heterotrimers that are endogenously formed. Furthermore, BLyS and APRIL appear to be differentially regulated [16,23-25], and their levels may even be inversely correlated [23], which is also suggested by a trend in our limited dataset of patient samples.

Differential TACI, BCMA, and BAFF-R receptor expression may favor biologic activity of the A_2_B versus the AB_2 _heterotrimers. The highest-affinity binding was observed between APRIL and BCMA-Ig, which was up to three orders of magnitude greater than observed for BLyS or heterotrimer binding to atacicept, BCMA-Ig, or BAFF-R-Ig. In the TACI-Jurkat assay, heterotrimer signaling was similar to that of APRIL homotrimers. The reduced potency of the heterotrimer ligands in the primary human B cell-proliferation assay compared with the homotrimeric BLyS may be explained by the predominant expression on circulating B cells of BAFF-R, to which our heterotrimers bind poorly owing to their predominantly A_2_B stoichiometry. Differences between receptor expression and/or B cell composition in the blood from different donors may explain the complex differences in dose-response curves observed in the primary B-cell assay.

Roschke *et al*. [6] also investigated the ability of soluble Ig fusion versions of TACI, BCMA, and BAFF-R to neutralize BLyS and BLyS/APRIL heterotrimers in a human B cell-proliferation assay. In that study, only TACI-Ig inhibited the BLyS/APRIL heterotrimer, and BCMA-Ig or BAFF-R-Ig was ineffective [6]. In our studies, both atacicept and BCMA-Ig neutralized the activity of BLyS, APRIL, and the recombinant BLyS/APRIL heterotrimer in the TACI-Jurkat and human B cell-proliferation assays, whereas BAFF-R-Ig inhibited BLyS but exhibited little or no inhibition of heterotrimer or APRIL activity. This was expected and consistent with the observed binding of BLyS, but not APRIL, to BAFF-R. It could be hypothesized that the Roschke *et al*. heterotrimers were composed of predominantly AB_2 _trimers; however, it is difficult to explain why these heterotrimers were not inhibited by soluble BAFF-R-Ig. This discrepancy would best be addressed by specifically purifying A_2_B and AB_2 _heterotrimers, and assessing their activity in the presence of each of the three soluble receptors. We would speculate that native A_2_B trimers are likely capable of binding to TACI and BCMA, whereas AB_2 _trimers should predominantly bind to TACI and possibly BAFF-R.

Soluble BLyS has been reported to form higher-order oligomers (for example, 60-mers) composed of multiple homotrimers, a cluster formation mediated by a flap-like region that is not present in APRIL [5]. Although some early reports showed that BLyS existed *in vivo *only in trimeric form [26,27], a more recent study suggested that BLyS 60-mers may form naturally *in vivo *and have biologic activity distinct from that of BLyS homotrimers [4]. However, no reports have been published of native oligomeric APRIL. It has been proposed that signaling through TACI in mature B cells or plasmablasts requires higher-order BLyS oligomers or the cross-linking of APRIL through its binding to proteoglycans, whereas BAFF-R and TACI on primary B cells can bind and respond to all forms of BLyS [4,8,13]. Our BLyS homotrimers signal less strongly than APRIL and the heterotrimers in the TACI-Jurkat assay, leading to a 20- to 25-fold difference in EC_50 _values between BLyS and APRIL or the heterotrimers. This finding appears to support the contention that BLyS oligomers may be required for optimal signaling through TACI. However, after extensive evaluation of the recombinant BLyS, APRIL, and heterotrimers using SEC-MALS and other techniques, we found no evidence for higher-order multimers or oligomerization of the ligands in this study (data not shown).

The inhibition of BLyS, APRIL, and the heterotrimers by atacicept is consistent with the observed effects of atacicept and/or murine TACI-Ig in preclinical and clinical studies. In mice and monkeys, atacicept reduces serum IgM levels and inhibits the IgM response to T-dependent antigen [28]. It inhibits B-cell maturation and survival, age-related T-cell activation, and the T cell-independent marginal zone B-cell response, and significantly decreases levels of plasma cells in the spleen and bone marrow [28-30]. However, atacicept does not reduce the numbers of B memory cells, which are active in long-term humoral immunity, as their survival is independent of BLyS or APRIL [31]. These biologic changes in response to atacicept are associated with reduced disease scores and prolonged survival in SLE-prone mice [19,29,30,32]. In Phase Ib studies, subcutaneous atacicept treatment reduced serum Ig, mature B-, and total B-cell levels in patients with RA or SLE [17,18]. These actions were coupled with promising exploratory effects on disease-activity measures [17,18,33]. Phase II/III trials are currently assessing the efficacy and tolerability of atacicept in patients with these conditions.

Our patient cohort data support and expand on a previous report showing higher serum levels of BLyS/APRIL heterotrimers in a limited sample of patients with autoimmune diseases (n = 15) compared with healthy controls (n = 6) [6]. Roschke *et al*. [6] investigated whether BLyS/APRIL heterotrimers are elevated in patients with autoimmune diseases, and reported levels of up to ~230 ng/ml [6]. In the Roschke *et al*. study, a mAb reagent capable of immunoprecipitating the heterotrimers was identified, and an assay was performed to measure heterotrimers by using an ELISA strategy. Data were reported from two separate ELISA assays, using either the anti-heterotrimer mAb or an anti-BLyS pAb as capture antibodies to quantify the proteins in patient sera. In several cases, data from the two assays differed by an order of magnitude for the same sample. The authors postulated that the higher heterotrimer levels were detected with the pAb assay because of better capture abilities than the mAb-based assay, or a possible preference for either the A_2_B or the AB_2 _forms of the heterotrimers. In contrast, the immunoassay described in the current study was designed to detect both the A_2_B and AB_2 _forms of the heterotrimers, and heterotrimer levels were quantified by using laser detection of fluorescently labeled detection mAbs. The results from our assay suggest that the levels of heterotrimers *in vivo*, even in very ill patients, are similar to or lower than those of the homotrimeric forms of BLyS and APRIL, with serum concentrations of native heterotrimers observed that were typically < 5 ng/ml. However, although heterotrimer levels are typically somewhat lower than those of the homotrimers, in certain patients, they may be found in similar or even greater concentrations. Larger studies with well-characterized assays are needed to assess accurately the relative levels of BLyS, APRIL, and heterotrimers, and to determine how common elevations are in their levels in clinical populations.

In the serum samples from patients with SLE and RA used in this study, levels of BLyS, APRIL, and heterotrimer were elevated in patients with SLE, compared with the sera of healthy donors. The detection of a single ligand (BLyS, APRIL, or heterotrimer) in more than one third of the samples may reflect specific control mechanisms for these TNF family members. The available data also suggest a trend toward correlation of BLyS and heterotrimer levels in patients with SLE, although this result is based on a very small number of samples with levels above the assay LOQ for both ligands.

It should be noted that, although our analysis shows that APRIL is detectable in a higher fraction of patients with SLE than in healthy controls, some controversy exists with regard to the role of APRIL in SLE. Several studies [6,14,24] have shown that APRIL levels are elevated in patients with SLE. Koyama *et al*. [14] showed a trend between APRIL levels and anti-dsDNA Ab levels and a correlation with the British Isles Lupus Assessment Group (BILAG) index score of musculoskeletal disease. In contrast, Stohl *et al*. [24] reported an inverse correlation between APRIL and anti-dsDNA Ab levels and disease activity measured by SLEDAI score. Another recent study reported high APRIL levels in the sera of patients with SLE, but no correlation with SLEDAI score [23]. The use of different APRIL assays may contribute to the disparate results currently in the literature, as some assays (including ours) show serum APRIL concentrations of 2-8 ng/ml (see, for example, Planelles 2004 (34)), whereas other assays yield much higher values, ≤ 2,500 ng/ml [14,16,23,35-37]. A possible explanation for these discrepancies is the biochemical characteristics of the recombinant APRIL used to generate capture and detection reagents and to provide reference standards for each assay. In our experience, some commercially available forms of APRIL, when used as reference standards, yield inaccurate (high) or imprecise determinations of native APRIL levels in sera (unpublished observations).

The present analysis of our limited RA patient cohort shows that APRIL may be specifically elevated in RA, whereas BLyS or heterotrimer levels do not appear to be increased. Others have previously reported that levels of both BLyS and APRIL in patients with RA are higher in synovial fluid than in serum, suggesting that these ligands play an important role in the inflamed synovial compartment [15,16,38]. It would be of interest to repeat these studies of matched RA serum and synovial fluid samples by using our APRIL assay.

Our analysis of the subset of serum samples from patients with SLE for which corresponding disease-activity data were available (that is, SLEDAI scores, anti-dsDNA Abs, C3 and C4 levels, and ESR) indicates that elevated heterotrimer levels may be associated with increasing SLE disease activity (Supplemental Tables 1 and 2 in Additional file 2). Thus, further studies with larger group sizes are warranted to pursue this possible correlation of heterotrimers with SLE disease activity. Indeed, a larger dataset should be constructed to confirm data trends identified in this study, and as disease levels may fluctuate for individual patients, information on disease activity scores (at the time of blood draw) should be collected, along with the serum BLyS, APRIL, and heterotrimer levels.

In agreement with previous reports [15], our data also show that BLyS is detectable in a higher fraction of patients with SLE than in healthy controls. BLyS levels also reportedly correlate with clinical disease activity in SLE [39] and RA [25]. Levels of BLyS have been shown to increase during anti-CD20 mAb-mediated B-cell depletion in patients with both SLE and RA, and to decline with B-cell repopulation in patients with SLE [25,40]. Similar elevations in BLyS have also been observed in sera from patients with Sjögren's syndrome and non-Hodgkin lymphoma treated with an anti-CD20 mAb [41,42]. In contrast, APRIL levels were reported to decrease during anti-CD20 mAb-mediated B-cell depletion in patients with SLE, whereas no significant changes in APRIL levels were observed in patients with RA undergoing anti-CD20 therapy [25]. To address the discrepancies in reported APRIL data from various laboratories, these studies are currently being repeated using our validated APRIL ELISA assay.

Given that heterotrimers have similar binding properties and *in vitro *activities to the BLyS and APRIL homotrimers, and may be present in sera in similar amounts to APRIL and BLyS, we postulate that they may likely play similar biologic roles to BLyS and APRIL in B-cell development and differentiation. As our recombinant A_2_B heterotrimers behave much like APRIL *in vitro*, we also speculate that AB_2 _heterotrimers would function more like BLyS, for example, playing a role in early B-cell survival and selection. BLyS exists in both soluble and transmembrane-bound forms, whereas APRIL is believed to exist only in a soluble form, with the exception of the TWEAK-APRIL fusion protein TWE-PRIL [43]. We did not test whether BLyS/APRIL heterotrimers were expressed on the cell surface, but if so, their potential for exerting biologic effects would presumably be expanded.

Whether native heterotrimers play a biologic role distinct from their homotrimeric counterparts remains to be determined. Our data suggest that investigating forms of BLyS and APRIL other than the conventional homotrimers in patients with autoimmune diseases may help to elucidate the pathology of such disorders and may also reveal additional disease markers and targets for treatment.

## Conclusions

Recombinant BLyS/APRIL heterotrimers are biologically active *in vitro *on TACI-transfected cells and on primary human B cells, and are inhibited by atacicept and BCMA-Ig. A new heterotrimer assay that detects both A_2_B and AB_2 _forms of BLyS/APRIL heterotrimers demonstrated that native heterotrimers are elevated in patients with SLE.

Further investigation of heterotrimer levels and their apparent relation with disease activity is warranted in a large cohort of patients with autoimmune diseases. Mechanistic studies to determine if BLyS/APRIL heterotrimers play a biologic role distinct from BLyS and APRIL, and whether native heterotrimers are inhibited by BLyS- and APRIL-targeting agents, would also be of interest.

## Abbreviations

APRIL: a proliferation-inducing ligand; BAFF: B cell-activating factor belonging to the tumor necrosis factor family; BAFF-R: BAFF receptor; BCMA: B cell-maturation antigen; BILAG: British Isles Lupus Assessment Group; BLyS: B-lymphocyte stimulator; CHO: Chinese hamster ovary; dsDNA: double-stranded DNA; EC_50_: 50% effective concentration; EDTA: ethylenediaminetetraacetic acid; ELISA: enzyme-linked immunosorbent assay; ESR: erythrocyte sedimentation rate; HAC: heparin affinity column; HRP: horseradish peroxidase; IC_50_: 50% inhibition concentration; Ig: immunoglobulin; K_D_: dissociation constant; LOQ: limit of quantitation; mAb: monoclonal antibody; NF: nuclear factor; otPA: optimized tissue plasminogen activator; pAb: polyclonal antibody; PBS: phosphate-buffered saline; PCR: polymerase chain reaction; RA: rheumatoid arthritis; SDS-PAGE: sodium dodecylsulfate polyacrylamide gel electrophoresis; SEC: size-exclusion chromatography; SEC-MALS: SEC with multiangle light scattering; SLE: systemic lupus erythematosus; SLEDAI: SLE Disease Activity Index; TACI: transmembrane activator and CAML interactor; TNF: tumor necrosis factor; TRAF: TNF receptor-associated factor; zz: "zipper" trimerization domain.

## Competing interests

SRD, TRB, NBH, MM, and SP are current employees and stockholders of ZymoGenetics, Inc. MDM, BH, KBL, HL, MML, CMK, JLE, and JAG are ZymoGenetics, Inc., stockholders and former employees. ZymoGenetics, Inc., has filed multiple patent applications on atacicept and BLyS/APRIL heterotrimers. KBE and MHW received payment from ZymoGenetics, Inc., and Merck Serono S.A.-Geneva (an affiliate of Merck KGaA, Darmstadt, Germany) for the serum samples they provided. MD declares that she has no competing interests.

## Authors' contributions

SRD coordinated the data collection, helped design and interpret the study, and co-wrote the manuscript. BH developed the heterotrimer assay, measured heterotrimer and BLyS levels in serum samples, and ran the heterotrimer signaling assays. KBL designed the Biacore studies, co-developed the trypsin cleavage method, and contributed to data analysis. MDM designed the heterotrimer expression vectors and co-invented the zz trimerization domain. HL performed early-stage heterotrimer purity assessment. TRB co-developed the heterotrimer purification process and strategy. NBH co-developed and implemented the heterotrimer purification process and the trypsin cleavage method. MML designed and performed the SEC-MALS analyses of the heterotrimer. MM ran the human B cell-proliferation assays. CMK performed the statistical analyses and correlation studies for the BLyS, APRIL, and heterotrimer serum levels in patients with SLE and RA, and co-wrote the manuscript. JLE helped design the heterotrimer assay and assisted with data analysis. SP coordinated the measurement of APRIL levels in serum samples and helped analyze data. KBE, MHW, and MD provided serum samples for analysis. JAG helped design the study and assisted with data analysis. All authors read and approved the final manuscript.

## Supplementary Material

Additional file 1 **Supplemental methods**. Additional methodologic details. Click here for file

Additional file 2 **Supplemental tables**. Supplemental Tables 1 and 2 show serum heterotrimer levels and disease-activity markers in a subset of samples from patients with SLE. Click here for file
