# Supplementary material for: B-lymphocyte stimulator/a proliferation-inducing ligand heterotrimers are elevated in the sera of patients with autoimmune disease and are neutralized by atacicept and B-cell maturation antigen-immunoglobulin
Source: Arthritis Res Ther. 2010 Mar 19;12(2):R48. doi: 10.1186/ar2959 (PMC2888197; doi:10.1186/ar2959)
Supplement: Additional file 1 — Supplemental methods. Additional methodologic details. [file ar2959-S1.PDF]

Supplementary materials and methods

Purification of recombinant BLyS/APRIL heterotrimers

Chinese hamster ovary (CHO)-conditioned media was concentrated 10× to make sample handling easier. Constituents in the CHO-conditioned media interfere with the binding of His-tagged proteins to immobilized metal affinity chromatography (IMAC) resin. Heterotrimers were, therefore, purified from CHO-conditioned media following buffer exchange into ZymoGenetics 1 × PBS (0.137 M NaCl, 0.0027 M KCl, 0.0072 M Na_2HPO_4, 0.0015 M KH_2PO4, pH 7.4) by ultrafiltration/diafiltration before loading onto IMAC resin. The concentrated buffer-exchanged media was captured on the IMAC resin, NI-NTA His Bind Superflow (Novagen, Gibbstown, NJ, USA) in batches. The resin binds all of the Hisx6-zz12.6 B-lymphocyte stimulator (BLyS) protein, with much of the Flag-zz12.6 a proliferation-inducing ligand (APRIL) homotrimer not captured.

The soluble ligands were purified further by sequential chromatography on a heparin affinity column (HAC) with gradient elution, followed by size exclusion chromatography (SEC). A HAC — Heparin AF-650M (Tosoh Bioscience, Montgomeryville, PA, USA) — was used to resolve zz12.6 heterotrimer from the remaining Flag-zz12.6 APRIL homotrimer. The heterotrimer eluted early in the increasing NaCl gradient, while APRIL eluted later. The eluate pool from the HAC was concentrated to <8 ml for injection over an SEC. The concentrated HAC pool was injected over a Superdex 200 Prep Grade Column (GE Healthcare, Piscataway, NJ, USA). The injection was always <3% of the volume of the column. SEC separated the heterotrimer from residual high molecular weight contaminants and aggregates and buffer exchanged the purified heterotrimer into the formulation buffer.

Generation and purification of non-tagged heterotrimers

To produce non-tagged heterotrimers, the Flag and His tags were cleaved from the Flag-zz12.6 form of APRIL and the Hisx6-zz12.6 form of BLyS by employing a limited proteolysis strategy. Trypsin was added to the buffered Flag-zz12.6 APRIL/Hisx6-zz12.6 BLyS heterotrimer at 1:100 ratio by mass. The combined materials were incubated for ~1 hour at 37ºC while reaction progress was monitored by reverse-phase high-performance liquid chromatography (RP-HPLC). The reaction was quenched via addition of 4-(2-aminoethyl) benzenesulfonyl fluoride hydrochloride to a final concentration of 0.56 mg/ml.

The reaction products were purified by sequential chromatography on a HAC, SEC, and a final batch process on immobilized anti-Flag tag resin to remove any non-cleaved product. Digested products were diluted from 250 mM NaCl to 150 mM NaCl using cold water and captured on a HAC (as above). Non-tagged heterotrimer was resolved from undigested products using a gradient of increasing NaCl concentration. Non-digested products eluted early in the gradient (150–350 mM) while the non-tagged product eluted later (350 mM to 1 M). The eluate pool was based on A280 nm inflection and analyzed via RP-HPLC. The HAC eluate pool containing non-tagged heterotrimer was then concentrated to <3 ml against 30 kD MWCO Ultracel membrane (Millipore, Billerica, MA, USA) and injected over an SEC column, 16/60 120 ml Superdex 200 (as above), using 50 mM $NaPO_4$, 250 mM NaCl, pH 7.2 as the mobile phase.

Any remaining tagged species was removed from the size exclusion pool via incubation with 1 ml of anti-Flag agarose resin (Sigma, St Louis, MO, USA) while slowly rocking overnight at 4°C. Flag resin equilibrated in the SEC mobile phase. The resin was separated from solution via 0.22 μm filtration and the filtrate was concentrated to 1 mg/ml.

N-terminal sequence analysis and SEC with multi-angle light scattering (SEC-MALS) analysis were consistent with both tags having been removed. Tag removal was also corroborated by the staining of western blots with antibodies specific for either tag.

Sodium dodecyl sulfate polyacrylamide gel electrophoresis analysis

APRIL or BLyS protein samples were mixed 1:1 with 2 × LDS sample buffer (Invitrogen, Carlsbad, CA, USA). Reduced samples were heated at 100°C for 8 minutes before loading 4–12% Bis-Tris gel. Non-reduced samples were not heated before loading 10% Bis-Tris gel. The gel was run at a constant 150 V for ~1 hour. For Coomassie staining, the gel was stained using Brilliant Blue R Staining Solution (Sigma-Aldrich, St Louis, MO, USA) diluted 1:4 in 10% acetic acid and 40% methanol solution.

Western blot analysis

Analysis by western blotting was performed using tag-specific antibodies to detect APRIL and BLyS chains separately. The gel was transferred to a 0.22 μm nitrocellulose membrane using a Hoeffer transfer apparatus (Hoeffer Scientific Instruments, San Francisco, CA, USA) according to the manufacturer's instructions. The membrane was blocked using non-fat dry milk (2.5% in block for Hisx6-zz12.6 anti-BLyS blot; 10% for all other blots) in ZymoGenetics western A buffer (0.10% [w/w] Tris base, 0.66% [w/w] Tris HCl, 0.19% [w/w] EDTA, 0.05% [v/w] IGEPAL®, 0.88% [w/w] NaCl, 0.25% [w/w] gelatine) for 20 minutes at room temperature. Antibody solutions were diluted in western A with non-fat dry milk (0% for Hisx6-zz12.6 anti-BLyS blot; 2.5% for all other blots) and the blot incubated for 1 hour at room temperature. Three 8-minute washes were performed in western A after antibody incubations and between primary and secondary incubations. The chemiluminescent signal was detected using Lumi Light Plus Western blotting

substrate (Roche, Indianapolis, IN, USA) and analyzed on the LumiImager system (Roche, Indianapolis, IN, USA).

Size-exclusion chromatography with multi-angle light scattering mass distribution analysis

The molecular mass of the heterotrimers was confirmed by SEC-MALS mass distribution LS/UV/RI 3-detector analysis using a 3-angle static light-scattering detector and differential refractometer (both from Wyatt Technology Corp., Santa Barbara, CA, USA) in line with a diode array UV detector (1100 HPLC; Agilent Technologies Inc., Santa Clara, CA, USA). SEC was performed with a Superdex-200 10 × 300 mm column in phosphate-buffered saline at a 0.4 ml/min flow rate from a 100 µg injection of protein. Data analysis was carried out with Wyatt Technology (Santa Barbara, CA, USA) ASTRA 5 software.

Binding kinetics and affinity studies

Binding affinities and kinetics of BLyS, APRIL, and heterotrimers for the receptor–Fc fusion proteins (atacicept, BCMA-Ig, and BAFF-R-Ig) were assessed via Biacore surface plasmon resonance studies using a Biacore 3000 analyzer equipped with Biacore Control and Evaluation software (version 3.2) (GE Healthcare, Piscataway, NJ, USA).

Atacicept, BCMA-Ig, and BAFF-R-Ig were covalently immobilized onto a Biacore CM4 sensor chip surface using a mixture of 0.4 M *N*-ethyl-*N*'-(3-diethylamino-propyl) carbodiimide and 0.1 M *N*-hydroxysuccinimide. Atacicept, BCMA-Ig, and BAFF R-Ig were diluted in 10 mM sodium acetate at pH 5.0, pH 5.5, or pH 5.5 and immobilized on flow cells 2, 3, and 4 to final levels of 223 resonance units (RU), 17 5 RU and 85 RU, respectively. Flow cell 1 was activated and blocked and used as the reference.

After immobilization, the remaining active sites on the flow cells were blocked with 1 M ethanolamine.

Binding studies were performed at 25ºC with a flow rate of 50 µl/min. Serial 1:2 dilutions of each ligand from approximately 0.05 to 20 nM were made in analysis buffer (20 mM sodium phosphate, 150 mM NaCl, 0.05% polysorbate 20, pH 7.5). The association time was 4 minutes, and the dissociation time was 10 minutes for the low concentration samples and 60 minutes for the two highest concentration samples. Injections were performed in random order with replicate injections for the buffer blanks and 3 of the 8 ligand concentrations. After each injection cycle the soluble receptor surface was regenerated with three 30 µl injections of 33 mM HCl at 50 µl/min.

The binding curves were processed by double referencing (subtraction of the signal from the reference flow cell followed by subtraction of the signal from the buffer injections). The binding curves were globally fitted to a 1:1 binding model. The stoichiometry of binding was not determined. Dissociation constant (K_D) values were determined from the kinetic rate constants (k_a and k_d).

Biologic activity assays

The ability of atacicept, BCMA-Ig, and BAFF-R-Ig to neutralize the activity of BLyS, APRIL, and heterotrimers was tested in both the transmembrane activator and CAML interactor (TACI)-Jurkat and human B cell proliferation assays. Activity and neutralization profiles were plotted, and the 50% effective (EC_{50}) and inhibitory (IC_{50}) concentrations were determined for the ligands in the absence or presence of atacicept, BCMA-Ig, or BAFF-R-Ig. Human B cell proliferation was assessed by pulsing the plates for 12–16 hours with ^{3}H-thymidine, then harvesting and counting the plates on Day 5.
