# Supplementary material for: B-lymphocyte stimulator/a proliferation-inducing ligand heterotrimers are elevated in the sera of patients with autoimmune disease and are neutralized by atacicept and B-cell maturation antigen-immunoglobulin
Source: Arthritis Res Ther. 2010 Mar 19;12(2):R48. doi: 10.1186/ar2959 (PMC2888197; doi:10.1186/ar2959)
Supplement: Additional file 2 — Supplemental tables. Supplemental Tables 1 and 2 show serum heterotrimer levels and disease-activity markers in a subset of samples from patients with SLE. [file ar2959-S2.PDF]

Supplemental Table 1. Individual values of serum heterotrimer levels and disease activity markers in a subset of patients with SLE.

SLE patient subgroup	Donor	HT (ng/ml)	SLEDAI[a]	Anti-dsDNA (IU/ml)	C3 (mg/dl)	C4 (mg/dl)	ESR (mm/hr)
Undetectable HT	1	<0.100	8	110	56	10	6
	2	<0.100	8	962	81	12	14
	3	<0.100	N/A	N/A	N/A	N/A	80
	4	<0.100	6	260	106	28	100
	5	<0.100	6	207	60	10	4
	6	<0.100	8	229	108	24	36
	7	<0.100	2	39	91	18	42
	8	<0.100	6	44	166	41	31
	9	<0.100	6	222	100	12	16
Low HT[b] (≤0.227 ng/ml)	10	0.129	8	150	82	20	62
	11	0.134	4	41	60	11	10
	12	0.138	8	374	69	10	12
	13	0.139	10	619	113	11	42
	14	0.148	10	545	45	10	90
	15	0.156	6	N/A	115	13	26
	16	0.160	18	43	106	19	96
	17	0.164	4	449	47	10	2
	18	0.174	4	868	46	10	102
	19	0.181	8	509	42	14	100
	20	0.181	12	660	43	10	91
	21	0.203	12	875	71	14	80

	22	0.207	12	72	72	16	12
	23	0.227	4	33	75	24	43
High HT[b] (>0.227 ng/ml)	24	0.244	6	417	73	10	53
	25	0.247	6	98	86	17	15
	26	0.250	4	N/A	74	16	8
	27	0.266	10	N/A	37	10	100
	28	0.270	12	679	59	11	15
	29	0.277	15	1400	38	10	60
	30	0.319	20	1856	76	10	61
	31	0.322	9	1332	55	15	98
	32	0.324	16	150	58	15	13
	33	0.412	2	32	98	28	62
	34	0.431	8	515	78	17	36
	35	0.443	28	19	42	10	54
	36	0.725	8	9032	52	10	100

[a]SLEDAI score at time of serum draw.

[b]Median serum heterotrimer level for the subset of samples from patients with SLE with detectable heterotrimers (n = 27) was 0.227 ng/ml. This median value was chosen to separate the “low” from the “high” heterotrimer expressing groups.

C3, C4: complement components; ds-DNA; double stranded DNA; ESR: erythrocyte sedimentation rate; HT: heterotrimer; N/A: not available; SLE: systemic lupus erythematosus; SLEDAI: SLE disease activity index.

Supplemental Table 2. Correlation of heterotrimer serum levels with disease activity markers in a subset of patients with SLE.

SLE patient subgroup	n	HT[a] (ng/ml)	SLEDAI[a,b]	Anti-dsDNA[a] (IU/ml)	C3[a] (mg/dl)	C4[a] (mg/dl)	ESR[a] (mm/hr)
Undetectable HT	9	0.100	6.25 ± 1.98	259.1 ± 296.6	96.0 ± 34.4	19.2 ± 11.1	36.6 ± 33.4
Low HT[c] (≤0.227 ng/ml)	14	0.167 ± 0.03	8.57 ± 4.11	402.9 ± 310.5	70.4 ± 25.8	13.7 ± 4.5	54.8 ± 38.1
High HT[c] (>0.227 ng/ml)	13	0.348 ± 0.133	11.08 ± 7.15	1411.8 ± 2603.1	63.5 ± 19.0	13.8 ± 5.2	51.9 ± 33.4

[a]Mean ± standard deviation.

[b]SLEDAI score at time of serum draw.

[c]Median serum heterotrimer level for the subset of samples from patients with SLE with detectable heterotrimers (n = 27) was 0.227 ng/ml. This median value was chosen to separate the “low” from the “high” heterotrimer expressing groups.

C3, C4: complement components; ds-DNA; double stranded DNA; ESR: erythrocyte sedimentation rate; HT: heterotrimer; N/A: not available; SLE: systemic lupus erythematosus; SLEDAI: SLE disease activity index.
